# Reliability and validity of the Thai version of the Rapid Mood Screener (RMS-T)

**DOI:** 10.1192/j.eurpsy.2026.11189

**Published:** 2026-07-31

**Authors:** C. Som-On, C. Suradom, S. Suttajit, S. Kawilapat, M. Srisurapanont

**Affiliations:** 1 Department of Psychiatry, Faculty of Medicine; 2 Research Administration Section, Faculty of Medicine, Chiang Mai university, Chiang Mai, Thailand

## Abstract

**Introduction:**

Bipolar disorder (BD) is frequently misdiagnosed as unipolar depressive disorders, resulting in adverse clinical consequences. The Rapid Mood Screener (RMS) was developed to improve BD detection. (McIntyre RS, et al. Curr Med Res Opin. 2021; 37, 135-144).

**Objectives:**

To translate the RMS into Thai (RMS-T) and evaluate its psychometric properties in Thai adults with Bipolar I (BD-I) and Bipolar II (BD-II) disorders.

**Methods:**

The RMS underwent forward translation, back-translation, and cultural adaptation. Outpatients with major depressive disorder (MDD; n = 111) and BD (n = 31; BD-I = 21, BD-II = 10) were enrolled. Psychometric properties included internal consistency, interrater reliability, content validity, and test–retest reliability (2-week interval in a sample of 30), and criterion validity against the Mini International Neuropsychiatric Interview, version 5 (MINI-5). Criterion validity of RMS-T versus the Mood Disorder Questionnaire (MDQ) was compared using receiver operating characteristics (ROC) area under the curve (AUC) analyses, and optimal cut-off were identified using Youden’s index.

**Results:**

Internal consistency was acceptable (Cronbach’s alpha = 0.612, P < 0.001; McDonald’s Omega = 0.633, P < 0.001). Interrater reliability was fair to moderate (Cohen’s Kappa = 0.326, P < 0.001). Content validity indices ranged from 0.36 to 0.75 (P < 0.001). Image 1 shows the ROC curves differentiating BD from non-BD (n = 111), AUC (95% CI; P): RMS-T = 0.798 (0.720 to 0.876; P < 0.001), T-MDQ = 0.823 (0.731 to 0.915; P < 0.001), and AUC difference = -0.02530 (−0.108 to 0.0573; P = 0.549). Image 2 shows ROC curves differentiating BD-I from non-BD-I (n = 121), AUC (95% CI, P): RMS-T = 0.755 (0.657 to 0.854; P < 0.001), T-MDQ = 0.802 (0.700 to 0.904; P < 0.001); and AUC difference = -0.0468 (−0.144 to 0.0507; P = 0.347). For BD-II, AUC (95% CI, P) of the ROC curves of RMS-T was 0.786 (0.669 to 0.902; P < 0.001). Optimal RMS-T cut-offs were ≥4 for BD and BD-I (sensitivity 64.52% and 61.90%; specificity 77.48% and 73.55%) and ≥3 for BD-II (sensitivity 90.0%, specificity 53.79%): Youden’s indices of 42, 35.45, and 43.79, respectively. Positive predictive values were 44.44% (BD), 28.89% (BD-I), and 12.86% (BD-II); negative predictive values were 88.66%, 91.75%, and 98.61%. Test–retest reliability was strong (Spearman’s rho = 0.855, P < 0.001).

**Image 1:**
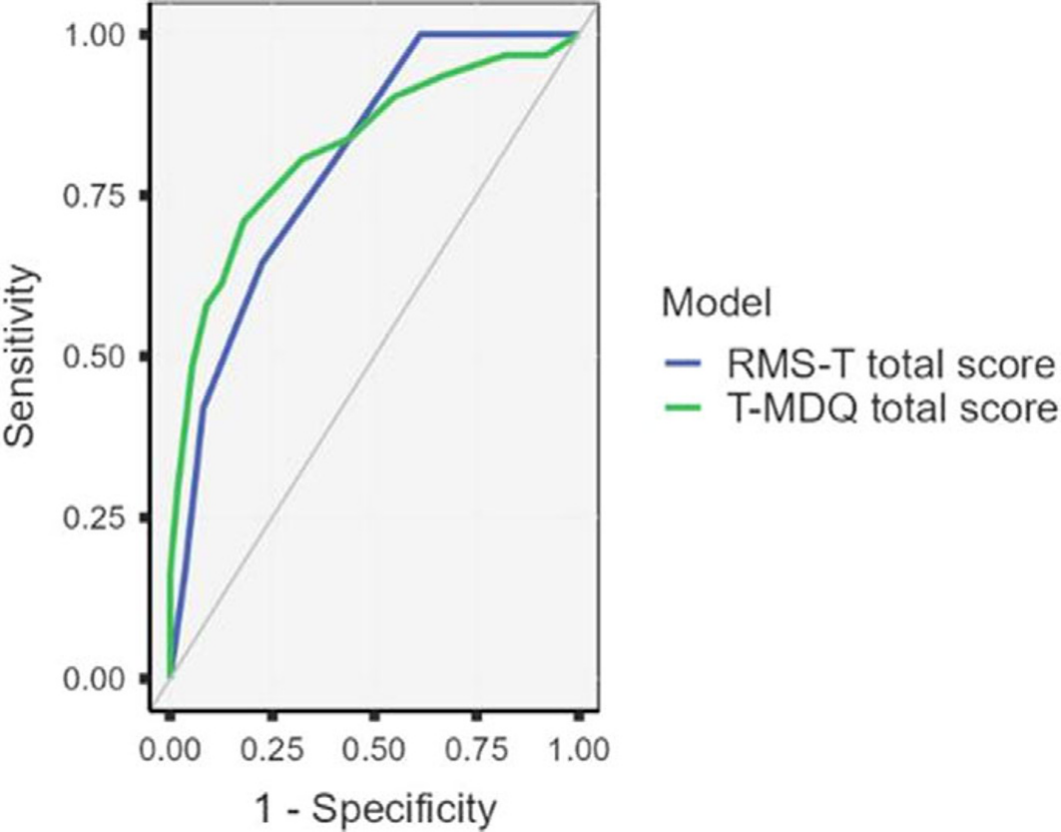
Image 1: Long description.

**Image 2:**
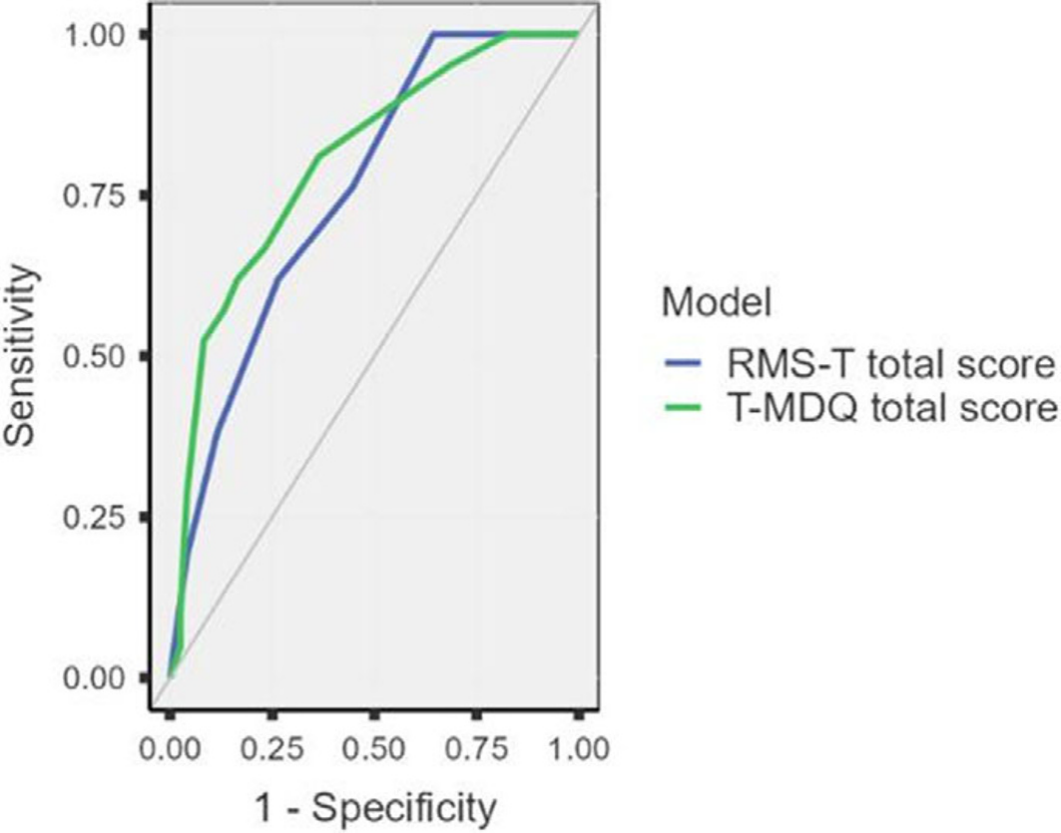
Image 2: Long description.

**Conclusions:**

The RMS-T is a valid, practical, time-efficient, six-item screening tool for identifying BD in patients presenting with a depressive episode. Further studies should assess its clinical utility and cost-effectiveness in boarder settings (e.g., primary care hospitals) and populations (e.g., older adults).

**Disclosure of Interest:**

None Declared

## Long descriptions

### Image 1: Long description

The x-axis is labeled 1 minus Specificity with values from 0.00 to 1.00. The y-axis is labeled Sensitivity with values from 0.00 to 1.00. A thin gray diagonal line extends from the origin 0,0 to the top-right 1,1 representing a random classifier.

Two data curves are plotted:

* The T-M D Q total score, shown as a green line, rises steeply from the origin, reaching a sensitivity of approximately 0.75 when 1 minus specificity is 0.25, then continues with a shallower slope toward 1,1.

* The R M S-T total score, shown as a blue line, rises less steeply initially compared to the green line but reaches a sensitivity of 1.00 earlier, at a 1 minus specificity value of approximately 0.60, where it plateaus horizontally until reaching the top-right corner.

A legend to the right of the plot identifies the blue line as R M S-T total score and the green line as T-M D Q total score.

Navigate back to Image 1:.

### Image 2: Long description

The line graph plots Sensitivity on the y-axis against 1 minus Specificity on the x-axis, both ranging from 0.00 to 1.00 in increments of 0.25. A thin gray diagonal line extends from the origin 0.00, 0.00 to the top-right corner 1.00, 1.00 as a baseline for chance performance.

Two data series are plotted:

* R M S dash T total score (blue line): This curve rises steeply from the origin, reaching a sensitivity of approximately 0.65 at 1 minus specificity of 0.25, then continues to a sensitivity of 1.00 at 1 minus specificity of approximately 0.60, where it plateaus.

* T dash M D Q total score (green line): This curve shows a more rapid initial ascent, reaching a sensitivity of 0.50 at 1 minus specificity of approximately 0.10. It then follows a more gradual concave path, crossing under the blue line at 1 minus specificity of 0.50, and reaching a sensitivity of 1.00 at 1 minus specificity of approximately 0.80.

A legend to the right of the plot identifies the blue line as R M S dash T total score and the green line as T dash M D Q total score.

Navigate back to Image 2:.

